# Supplementary material for: COZOID: contact zone identifier for visual analysis of protein-protein interactions
Source: BMC Bioinformatics. 2018 Apr 6;19:125. doi: 10.1186/s12859-018-2113-6 (PMC5889581; doi:10.1186/s12859-018-2113-6)
Supplement: Supplementary file 2 — Software build. Executable binary file of the software tool. (ZIP 118,784 kb) [file 12859_2018_2113_MOESM2_ESM.zip › cozoid/java/docs/beansbinding-1.2.1-doc.zip!/index-files/index-6.html]

F-Index (Beans Binding)


---


|  |  |  |
| --- | --- | --- |
| **Overview** \| Package \| Class \| Use \| **Tree** \| **Deprecated** \| **Index** \| **Help** | |  |
| **PREV LETTER**   **NEXT LETTER** | **FRAMES**    **NO FRAMES**     **All Classes** |  |


A B C D E F G H I J K L M N O P Q R S T U V W 

---


## **F**

**failed()** - Method in class org.jdesktop.beansbinding.Binding.ValueResult: Returns `true` if this `ValueResult` represents a failure and `false` otherwise. **FALSE** - Static variable in interface org.jdesktop.el.impl.parser.ELParserConstants: **FillBuff()** - Method in class org.jdesktop.el.impl.parser.SimpleCharStream: **fireElementChanged(int)** - Method in class org.jdesktop.observablecollections.ObservableCollections.ObservableListHelper: Sends notification that the element at the specified index has changed. **firePropertyChange(String, Object, Object)** - Method in class org.jdesktop.beansbinding.Binding: Sends a `PropertyChangeEvent` to the `PropertyChangeListeners` registered on the `Binding`. **firePropertyChange(Object, Object)** - Method in class org.jdesktop.swingbinding.adapters.BeanAdapterBase: **firePropertyStateChange(PropertyStateEvent)** - Method in class org.jdesktop.beansbinding.PropertyHelper: Notify listeners that the state of this property has changed, as characterized by the given `PropertyStateEvent`. **FLOATING\_POINT\_LITERAL** - Static variable in interface org.jdesktop.el.impl.parser.ELParserConstants: **FloatingPoint()** - Method in class org.jdesktop.el.impl.parser.ELParser: **forName(String)** - Static method in class org.jdesktop.el.impl.util.ReflectionUtil: **forNamePrimitive(String)** - Static method in class org.jdesktop.el.impl.util.ReflectionUtil: **Function()** - Method in class org.jdesktop.el.impl.parser.ELParser: **FunctionMapper** - Class in org.jdesktop.el: The interface to a map between EL function names and methods. **FunctionMapper()** - Constructor for class org.jdesktop.el.FunctionMapper: **FunctionMapperFactory** - Class in org.jdesktop.el.impl.lang: **FunctionMapperFactory(FunctionMapper)** - Constructor for class org.jdesktop.el.impl.lang.FunctionMapperFactory: **FunctionMapperImpl** - Class in org.jdesktop.el.impl.lang: **FunctionMapperImpl()** - Constructor for class org.jdesktop.el.impl.lang.FunctionMapperImpl: **FunctionMapperImpl.Function** - Class in org.jdesktop.el.impl.lang: **FunctionMapperImpl.Function(String, String, Method)** - Constructor for class org.jdesktop.el.impl.lang.FunctionMapperImpl.Function: **FunctionMapperImpl.Function()** - Constructor for class org.jdesktop.el.impl.lang.FunctionMapperImpl.Function: **functions** - Variable in class org.jdesktop.el.impl.lang.FunctionMapperImpl: **FUNCTIONSUFFIX** - Static variable in interface org.jdesktop.el.impl.parser.ELParserConstants

---


|  |  |  |
| --- | --- | --- |
| **Overview** \| Package \| Class \| Use \| **Tree** \| **Deprecated** \| **Index** \| **Help** | |  |
| **PREV LETTER**   **NEXT LETTER** | **FRAMES**    **NO FRAMES**     **All Classes** |  |


A B C D E F G H I J K L M N O P Q R S T U V W 

---
